# The definition of major trauma using different revisions of the abbreviated injury scale

**DOI:** 10.1186/s13049-021-00873-7

**Published:** 2021-05-27

**Authors:** Jan C. Van Ditshuizen, Charlie A. Sewalt, Cameron S. Palmer, Esther M. M. Van Lieshout, Michiel H. J. Verhofstad, Dennis Den Hartog, Dutch Trauma Registry Southwest, N. M. R. Soesman, T. S. C. Jakma, M. Waleboer, M. Staarink, M. M. M. Bruijninckx, A. Y. M. V. P. Cardon, P. T. den Hoed, G. R. Roukema, C. H. van der Vlies, N. W. L. Schep, L. van de Schoot

**Affiliations:** 1 Department of Surgery, Trauma Research Unit, Erasmus MC, University Medical Center Rotterdam, P.O. Box 2040, 3000 CA Rotterdam, The Netherlands; 2 Department of Public Health, Erasmus University Medical Centre Rotterdam, Rotterdam, The Netherlands; 3 Department of Epidemiology & Preventive Medicine, Monash University, Melbourne, Australia; 4 Trauma Service, Royal Children’s Hospital Melbourne, Parkville, Australia; 5 Department of Surgery, Francisus Gasthuis & Vlietland, Rotterdam, the Netherlands; 6 Department of Surgery, Albert Schweitzer Hospital, Dordrecht, the Netherlands; 7 Department of Surgery, Admiraal de Ruyter Hospital, Goes, the Netherlands; 8 Department of Surgery, Het Van Weel-Bethesda Hospital, Dirksland, the Netherlands; 9 Department of Surgery, IJsselland Hospital, Capelle aan den IJssel, the Netherlands; 10 Department of Surgery, ZorgSaam Zeeuws-Vlaanderen Hospital, Terneuzen, the Netherlands; 11 Department of Surgery, Ikazia Hospital, Rotterdam, the Netherlands; 12 Department of Surgery, Maasstad Hospital, Rotterdam, the Netherlands; 13 Burn Center, Maasstad Hospital, Rotterdam, the Netherlands; 14 Department of Surgery, Spijkenisse Medisch Centrum Hospital, Rotterdam, the Netherlands; 15 Department of Surgery, Beatrixhospital, Gorinchem, the Netherlands

**Keywords:** ‘AIS’, ‘ISS’, ‘Major trauma’, ‘In-hospital mortality’, ‘Quality indicator’

## Abstract

**Background:**

A threshold Injury Severity Score (ISS) ≥ 16 is common in classifying major trauma (MT), although the Abbreviated Injury Scale (AIS) has been extensively revised over time. The aim of this study was to determine effects of different AIS revisions (1998, 2008 and 2015) on clinical outcome measures.

**Methods:**

A retrospective observational cohort study including all primary admitted trauma patients was performed (in 2013–2014 AIS98 was used, in 2015–2016 AIS08, AIS08 mapped to AIS15). Different ISS thresholds for MT and their corresponding observed mortality and intensive care (ICU) admission rates were compared between AIS98, AIS08, and AIS15 with Chi-square tests and logistic regression models.

**Results:**

Thirty-nine thousand three hundred seventeen patients were included. Thresholds ISS08 ≥ 11 and ISS15 ≥ 12 were similar to a threshold ISS98 ≥ 16 for in-hospital mortality (12.9, 12.9, 13.1% respectively) and ICU admission (46.7, 46.2, 46.8% respectively). AIS98 and AIS08 differed significantly for in-hospital mortality in ISS 4–8 (χ^2^ = 9.926, *p* = 0.007), ISS 9–11 (χ^2^ = 13.541, *p* = 0.001), ISS 25–40 (χ^2^ = 13.905, *p* = 0.001) and ISS 41–75 (χ^2^ = 7.217, *p* = 0.027). Mortality risks did not differ significantly between AIS08 and AIS15.

**Conclusion:**

ISS08 ≥ 11 and ISS15 ≥ 12 perform similarly to a threshold ISS98 ≥ 16 for in-hospital mortality and ICU admission. This confirms studies evaluating mapped datasets, and is the first to present an evaluation of implementation of AIS15 on registry datasets. Defining MT using appropriate ISS thresholds is important for quality indicators, comparing datasets and adjusting for injury severity.

**Level of evidence:**

Prognostic and epidemiological, level III.

**Supplementary Information:**

The online version contains supplementary material available at 10.1186/s13049-021-00873-7.

## Background

The Abbreviated Injury Scale (AIS) [1–4] provides an anatomically-based, mortality-weighted code set used to classify injury severity. AIS coding, and AIS-derived scores such as the Injury Severity Score (ISS) [5, 6] are used to identify and classify injured patients within trauma systems, and can also be used as a component of risk adjustment and benchmarking using mortality prediction models [7–11]. Although based on expert opinion, the ISS has persevered for over 40 years as the ‘gold standard’ [8, 12] of injury scoring. The ISS is commonly used to define major trauma (MT) using an ISS ≥ 16. This threshold was adopted following evaluation of mortality rates in the North American Major Trauma Outcome Study in the 1980s [13, 14]. However, in recent years focus on outcome measures in trauma care has shifted from fatal to nonfatal outcomes [15–18]. Also, the ISS has substantial limitations, including in the prediction of outcome after serious injury [8, 10, 11, 19–21].

AIS code sets are periodically revised to better reflect contemporary performance of trauma systems. Not all trauma registries use the same AIS revision at any given time; worldwide, registries implement newer AIS revisions whenever considered necessary at a local level. However, differences in the classification of injury severity between AIS revisions can affect assessments of injury severity, both in individual patients and across populations. In turn, this can compromise assessments of quality of care, or of the level of performance of trauma systems.

In particular, changing from the widely used 1998 AIS update [3] (AIS98) to the 2008 update [2] (AIS08) profoundly affects descriptions of injury severity [22, 23] and outcome predictions [4, 24]. In AIS08, many injuries were re-assigned to higher or lower severity levels, although in practice more injuries decreased in severity [25]. As a result, the distribution of the ISS has down-shifted causing a 20% decrease in MT patients [26]. In addition, an increase in mortality rate, length of stay (LOS), need for intensive care (ICU) and urgent surgery has been reported in (re-classified) MT populations due to this shift [25, 27]. This affects measurements of the performance of trauma care over time, or across registries using different AIS revisions. The effects of a subsequent AIS revision in 2015 (AIS15) [4] on a trauma registry has not been evaluated in any published work.

Since AIS revisions can affect assessments of injury severity, it is of vital importance that the derived ISS is used to classify MT [27], particularly as other measures such as quality indicators rely heavily on such a classification [28]. The definition of MT becomes particularly relevant if a newer AIS revision is being implemented in a trauma registry, or if the time span of a study overlaps different AIS revisions. The present study aimed to assess the effects of different AIS revisions (and derived ISS) on clinical outcome measures and the volume of primary admitted MT patients to a designated regional level I trauma center.

## Methods

### General setting

The Dutch National Trauma Registry (DNTR) is nationally coordinated through 11 trauma regions. Yearly around 80,000 incidents are registered, of which approximately 5 % is considered as major trauma [29]. All patients admitted to the emergency department (ED) (within 48 h after trauma), followed by either hospitalization, transfer to other hospitals or death are included, excluding deaths on arrival. Trauma Region Southwest Netherlands (DTR SW) consists of urban, rural, industrial and tourist areas with a strong infrastructure, inhabited by 2.5 million people. Helicopter Emergency Medical Services (HEMS) are available. Each year around 10,000 inclusions are registered in the DTR SW, a region containing one level I trauma center. The proportion of primary admitted MT patients to a regional designated level I trauma centre (TC) is currently regarded as a quality indicator in the Netherlands.

### Population

Trauma patients were retrospectively selected from the DTR SW cohort between 2013 and 2016. AIS98 was used for injury coding until 2014, and from 2015 onward AIS08 was used. A wide variety of trauma settings and injuries are represented in the database, from traffic accidents or falls in private and leisure settings to burns, violence, drowning, asphyxia (hanging) and other forms of self-harm. The structure of the DTR SW trauma system did not change during the study period.

### AIS comparison

AIS98 [3] was used for injury coding in 2013–2014; injuries from 2015 to 2016 were coded using AIS08 [2]. For the latter period, AIS08 codes were mapped to AIS15 using the tables provided in the AIS15 revision [4]. ISS was calculated from AIS codes for all patients; these were termed ISS98, ISS08 and ISS15 depending on the AIS revision from which they were derived.

### Comparison VSTR

For international comparison, and as a sensitivity analysis, the inclusion and exclusion criteria of the Victorian State Trauma Registry (VSTR) were fitted on the DTR SW database, as the DTR has more general inclusion criteria than the VSTR. The VSTR includes patients with an ISS ≥ 12, death in ED or after hospitalization, patients in need of urgent surgery or ICU > 24 h with mechanical ventilation, or a length of stay (LOS) greater than 3 days. Specific VSTR inclusion and exclusion criteria are available elsewhere [30].

### Data analysis

Patients who were transferred from one ED to another were identified, and records from the transferring hospital were excluded in order to avoid double-counting.

Cumulative in-hospital mortality rates above all possible ISS thresholds were calculated, and compared between AIS98, AIS08 and AIS15. The baseline in-hospital mortality threshold for MT was set at the in-hospital mortality rate of ISS98 ≥ 16. At the same threshold of ISS98 ≥ 16, the ICU admission rate was calculated. For the AIS08 and AIS15 revisions, new ISS thresholds were selected based on the in-hospital mortality and ICU admission rates of ISS98 ≥ 16 [27, 31].

For time periods 2013–2014 and 2015–2016, in which different AIS revisions (AIS98 and AIS08 respectively) were used for coding injuries, normality of distribution for continuous variables was tested using the Shapiro-Wilk test. All continuous variables were non-normally distributed. Descriptive statistics were reported as a median (P_25_-P_75_) for continuous variables and number (percentage) for categorical variables. A Mann-Whitney test was used when comparing two groups, and a Kruskal-Wallis test was used when comparing multiple groups. For nominal variables, a χ^2^-test or Fisher’s (two-sided) exact test was used as applicable. A *p*-value of 0.05 was considered significant.

Statistical differences in the distributions of ISS98 and ISS08, and ISS98 and ISS15 were calculated using a Mann-Whitney test, and the differences between ISS08 and ISS15 with a Wilcoxon Signed Rank test, as these measures were mapped and hence not independent. Statistical differences of in-hospital mortality within each ISS category between AIS revisions were tested with a χ^2^-test.

Logistic regression analysis was performed with in-hospital mortality as the outcome parameter, and the AIS revision and ISS considered as factors. Grouped ISS was checked for interaction with AIS revision. Odds ratios were calculated using logistic regression models (with 95% confidence intervals) for the association between MT (ISS ≥ 16) and in-hospital mortality, as well as for the new MT ISS thresholds and in-hospital mortality for AIS98 (2013–2014), AIS08 (2015–2016) and mapped AIS08 to AIS15 (2015–2016). Homogeneity of odds ratios, after stratifying for AIS revision, were tested with Breslow D statistics for AIS08 compared with AIS98 and AIS15 compared with AIS98. Statistical differences between OR’s for AIS08 and AIS15 compared with AIS98 were calculated with a Cochran-Mantel-Haenszel test.

This study was exempted by a local Medical Research Ethics Committee after being assessed as not subject to the Medical Research Involving Human Subjects Act due to the use of retrospective data. Strengthening the Reporting of Observational Studies in Epidemiology (STROBE) guidelines were followed. Statistical analyses were done with Statistical Package for Social Sciences version 24.0.0.0 (SPSS, Chicago, IL) and R software environment (version 3.2.2 or higher, the R Foundation for Statistical Computing, Vienna, Austria).

## Results

**Fig. 1:**
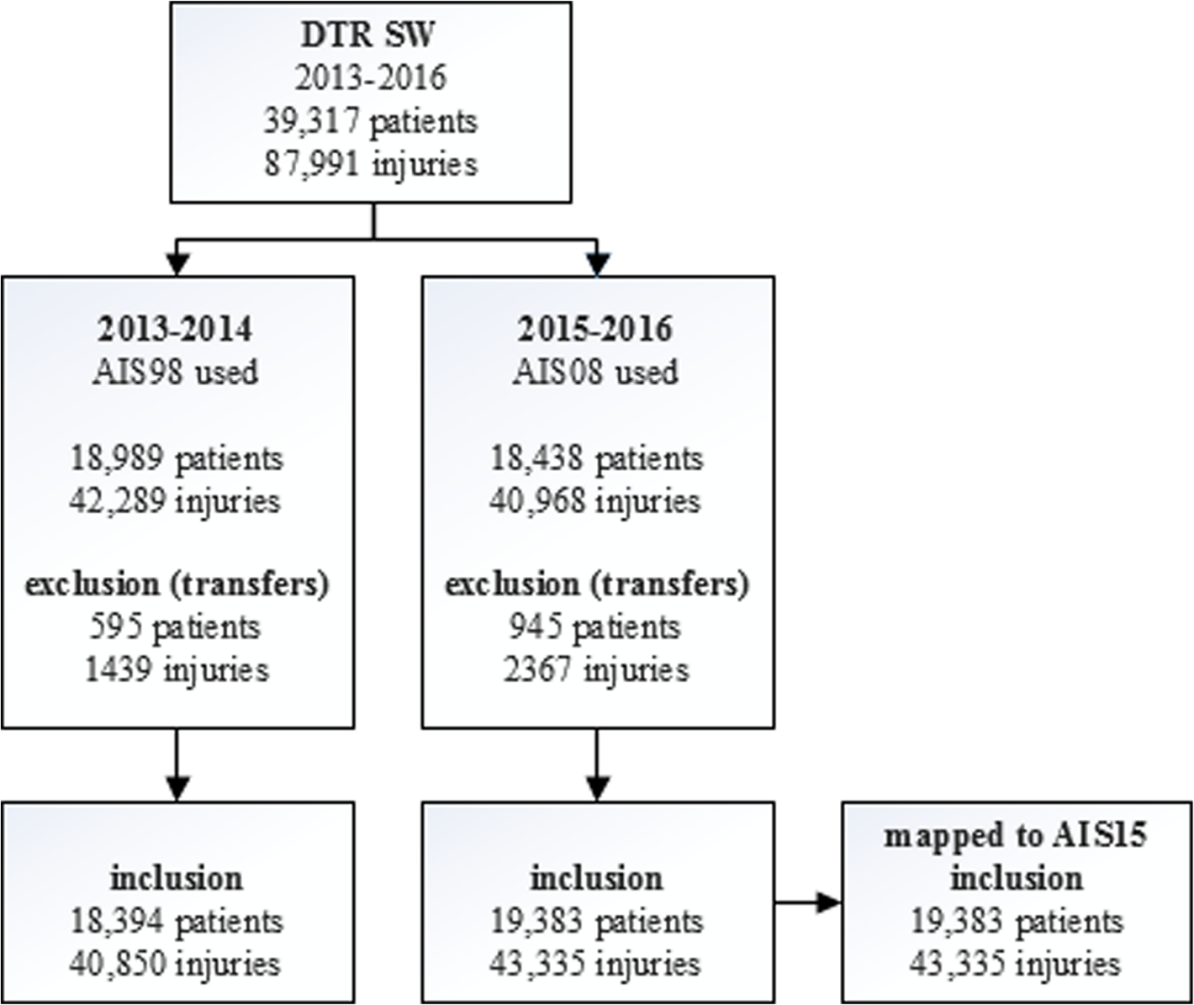
Inclusion flow chart

Records of 39,317 patients with a total of 87,991 injuries between 2013 and 2016 were registered in the DTR SW in the period 2013–2016; after excluding transfers between ED’s, 37,777 patients (84,185 injuries) were evaluated (Fig. 1). The 19,383 patients initially coded using AIS08 sustained 43,335 injuries; all of these were mapped to AIS15.

Between the two time periods in which different AIS revisions were used, the trauma populations were comparable (Table 1). Gender, age, injury mechanism, hospital length of stay in days (LOS), the number of days in an intensive/high/medium care unit (collectively termed ‘LOS ICU’) and number of days mechanically ventilated (LOS MV) did not differ significantly between the two time periods.

**Table 1:** Epidemiological comparison between DTR SW trauma populations of 2013-2014 and 2015-2016

		2013–2014 AIS98 used (***n*** = 18,394)	2015–2016 AIS08 used (***n*** = 19,383)	*p*
**Gender**	(male)	9123 (49.6%)	9630 (49.7%)	0.869
**Age**	(years)	60.9 (33.6–88.8)	61.2 (33.1–89.2)	0.238
**LOS**	(days)	3 (1–6)	3 (1–6)	0.086
**LOS ICU**	(days)	2 (1–4)	2 (1–4)	0.054
**LOS MV**	(days)	3 (1–8)	3 (1–8)	0.708
**Cause**^**a**^	Violence	299 (3.2%)	631 (3.3%)	0.752
	Traffic	1896 (20.2%)	3831 (19.9%)	0.388
	Work	378 (4.0%)	783 (4.1%)	0.959
	Home/Leisure	6122 (65.2%)	12,700 (65.6)	0.612
	Sport	567 (6.0%)	1103 (5.7%)	0.0234
	self-harm	89 (0.9%)	233 (1.2%)	0.055
	other	15 (0.2%)	16 (0.1%)	0.061

*LOS* Length Of Stay, *ICU* combination of admission to an ICU (Intensive Care Unit), High Care Unit (HCU); or Medium Care Unit (MCU), *MV* Mechanical Ventilation

^a^Cause was registered from 2014 onwards (9351 of 18,394 patients in 2013–2014). Statistical comparison is between 2014 (*n* = 21, cause unknown) and 2015–2016 (*n* = 86, cause unknown)

Medians (P_25_-P_75_) for ISS98, ISS08 and ISS15 were 9 (4–9), 5 (3–9) and 5 (4–9) respectively. There were significant differences between the distributions of ISS98, and both ISS08 (*U =* − 31.011*, p <* 0.0001) and ISS15 (*U =* − 16.112*, p <* 0.0001). A significant difference was also found between the distribution of the ISS15 compared to ISS08 (*Z* = − 55.693, *p <* 0.0001). The modified DTR SW data fitted by VSTR criteria resulted in medians (P_25_-P_75_) of 9 (6–16), 9 (5–14) and 9 (6–14) for ISS98, ISS08 and ISS15 respectively.

**Fig. 2:**
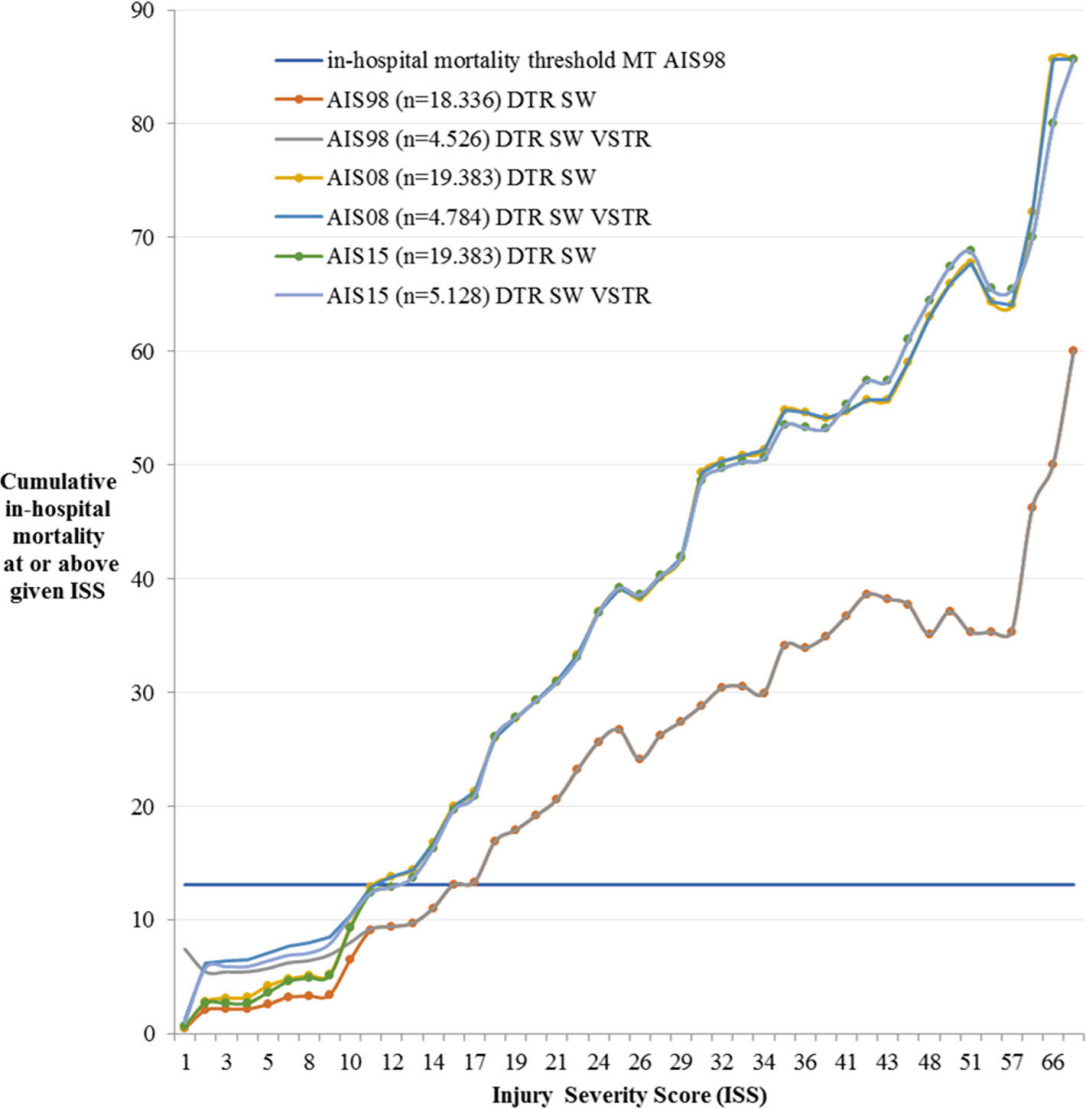
Cumulative in-hospital mortality for each ISS in the DTR SW and DTR SW with inclusion criteria of the VSTR, using AIS98, AIS08 and AIS15. For reference, the in-hospital mortality level of MT coded with AIS98 during 2013–2014 (13.1%) is indicated by the horizontal solid line

Cumulative in-hospital mortality levels at ISS ≥ 16 coded with AIS98, AIS08 and AIS15 were 13.1, 20.0 and 19.7% respectively (Fig. 2). The use of an ISS08 ≥ 11 and ISS15 ≥ 12 provided an equivalent in-hospital mortality risk. These differences between AIS revisions were also seen for the DTR SW population with the use of VSTR in- and exclusion criteria.

Dichotomised in-hospital mortality levels and ICU admission rates are presented in Table 2. In-hospital mortality rates with MT thresholds using an ISS ≥ 16 for all AIS revisions differed substantially. When an ISS ≥ 16 was used for all three AIS revisions, there was significant heterogeneity between revisions in terms of in-hospital mortality risk (Breslow-D, *p* = 0.025, Table S1). However, when odds ratios were compared with alternative MT thresholds across the three AIS revisions, Breslow-D statistics were non-significant, demonstrating homogeneity for comparisons between AIS98 and both AIS08 and AIS15. When adjusted MT thresholds were applied to the need for ICU, similar patient proportions were seen (Table 2).

**Table 2:** Contingency tables of in-hospital mortality and need for ICU for MT patients for AIS98, AIS08 and AIS15. MT is considered ISS ≥ 16 in the upper three contingency tables, the lower two rows of contingency tables utilize alternative ISS thresholds for MT (AIS08 ≥ 11, AIS15 ≥ 12). Percentages are row orientated. ICU, combination of admission to an ICU (Intensive Care Unit), High Care Unit (HCU); or Medium Care Unit (MCU)

**In-hospital mortality**											
**Using an ISS98 ≥ 16 threshold**				**Using an ISS08 ≥ 16 threshold**				**Using a ISS15 ≥ 16 threshold**			
	Died	Survived	Total		Died	Survived	Total		Died	Survived	Total
ISS < 16	208	16,912	17,120	ISS < 16	266	18,024	18,290	ISS < 16	267	18,009	18,276
	*1.2%*	*98.8%*			*1.5%*	*98.5%*			*1.5%*	*98.5%*	
ISS ≥ 16	159	1057	1216	ISS ≥ 16	219	874	1093	ISS ≥ 16	218	889	1107
	*13.1%*	*86.9%*			*20.0%*	*80.0%*			*19.7%*	*80.3%*	
Total	367	17,969	18,336	Total	485	18,898	19,383	Total	485	18,898	19,383
				**Using an ISS08 ≥ 11 threshold**				**Using a ISS15 ≥ 12 threshold**			
					Died	Survived	Total		Died	Survived	Total
				ISS08 < 11	258	17,363	17,621	ISS15 < 12	259	17,375	17,634
					*1.5%*	*98.5%*			*1.5%*	*98.5%*	
				ISS08 ≥ 11	227	1535	1762	ISS15 ≥ 12	226	1523	1749
					*12.9%*	*87.1%*			*12.9%*	*87.1%*	
				Total	485	18,898	19,383	Total	485	18,898	19,383
**Need for ICU**											
**Using an ISS98 ≥ 16 threshold**				**Using an ISS08 ≥ 11 threshold**				**Using a ISS15 ≥ 12 threshold**			
	ICU	No ICU	Total		ICU	No ICU	Total		ICU	No ICU	Total
ISS < 16	1008	16,112	17,120	ISS08 < 11	1067	16,554	17,621	ISS15 < 12	1083	16,551	17,634
	*5.9%*	*94.1%*			*6.1%*	*93.9%*			*6.1%*	*93.9%*	
ISS ≥ 16	568	648	1216	ISS08 ≥ 11	824	938	1762	ISS15 ≥ 12	808	941	1749
	*46.7%*	*53.3%*			*46.8%*	*53.2%*			*46.2%*	*53.8%*	
Total	1.576	16,760	18,336	Total	1891	17,492	19,383	Total	1891	17,492	19,383

**Fig. 3:**
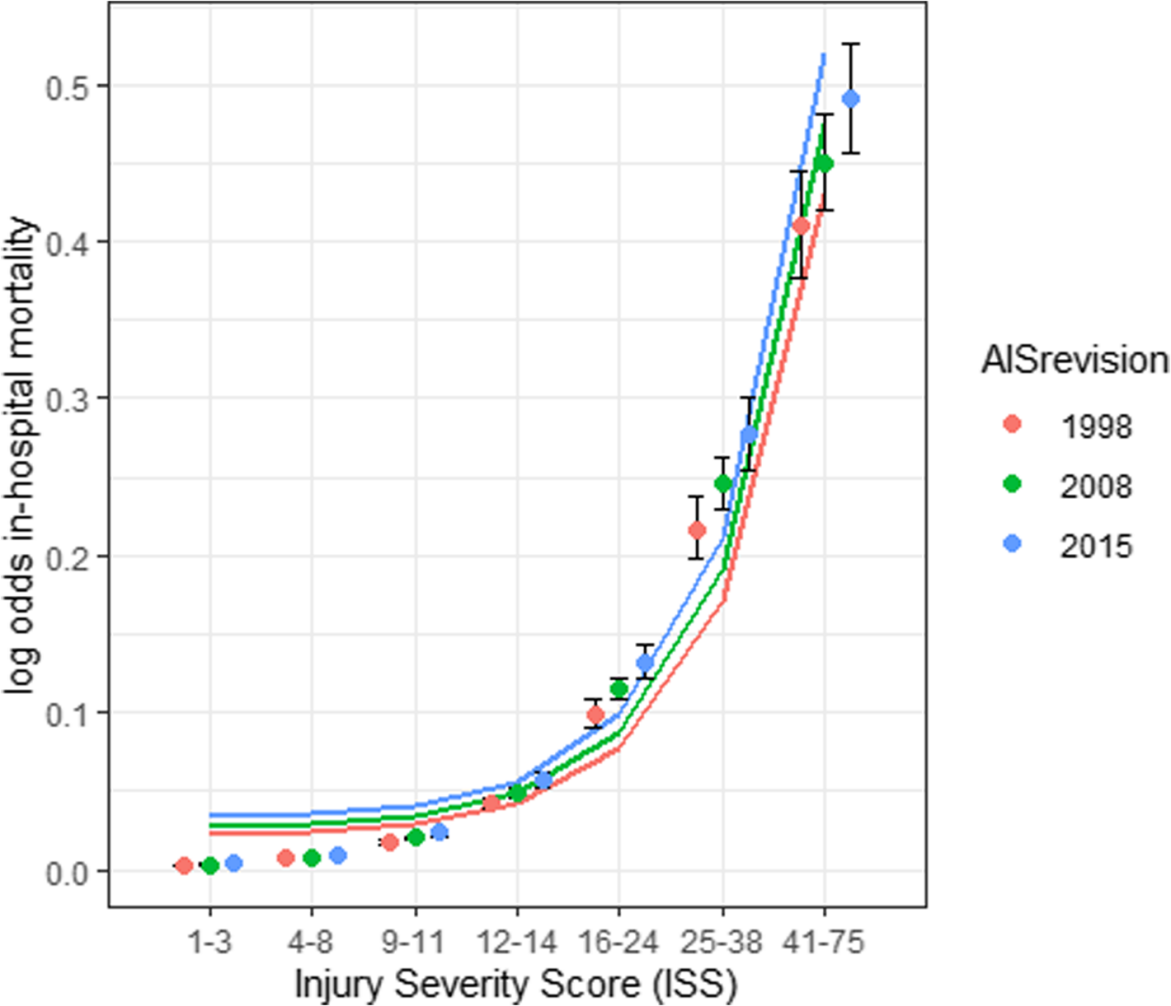
In-hospital mortality likelihood logistic regression for ISS categories and AIS revision. 95% confidence intervals for log odds are shown for each ISS category

Increasing ISS category resulted in an increased likelihood of death (Fig. 3 and Table S2). Logistic regression with AIS revision and ISS category as factors, and in-hospital mortality as an outcome resulted in a crude OR of 1.26 *(95% CI 1.096–1.441)* for AIS08. Significant in-hospital mortality differences between AIS revisions were seen for ISS 4–8 (χ^2^ = 9.926, *p* = 0.007), ISS 9–11 (χ^2^ = 13.541, *p* = 0.001), ISS 25–40 (χ^2^ = 13.905, *p* = 0.001) and ISS 41–75 (χ^2^ = 7.217, *p* = 0.027). No significant differences in in-hospital mortality risk were reported for any ISS category when comparing ISS calculated using AIS08 and AIS15.

**Fig. 4:**
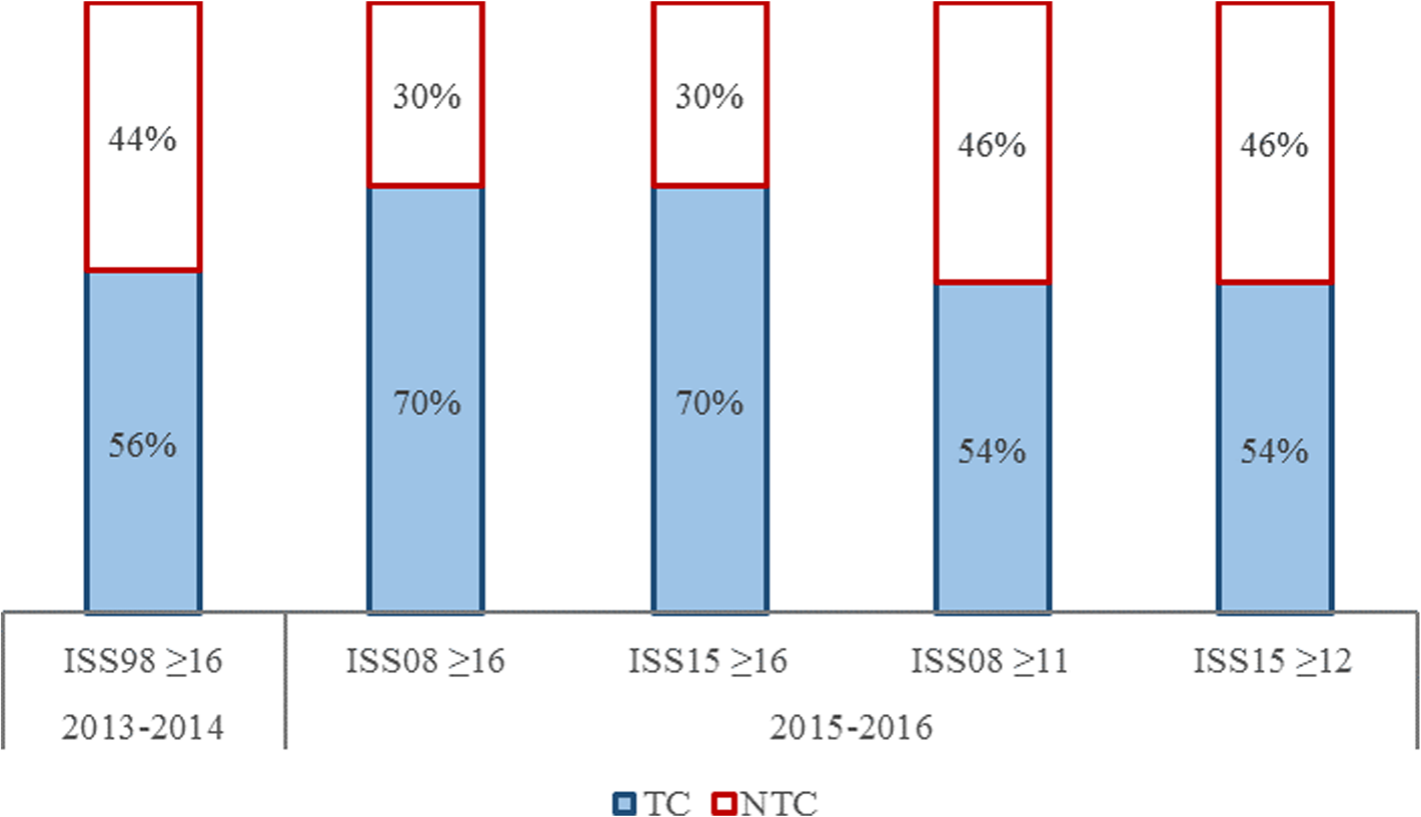
Allocation of MT patients in 2013–2016 in trauma region Southwest Netherlands (DTR SW), showing the proportions of MT patients taken directly to the designated TC (level I) or to NTC (non-level I). MT is considered to be ISS ≥ 16; alternative MT thresholds are shown for AIS08 (ISS ≥ 11) and AIS15 (ISS ≥ 12)

Figure 4 shows the proportions of primary admitted MT patients for DTR SW; 56% of MT in 2013–2014, and 70% in 2015–2016 arrived directly to the level 1 trauma center. With a threshold for MT at ISS08 ≥ 11 coded with AIS08, this percentage decreased to 54% in 2015–2016. Using a threshold for MT at ISS ≥ 12 with AIS15, the proportion of primary admitted MT patients remained at 54% in 2015–2016.

## Discussion

This is the first study in the world to report on the potential effects of adopting AIS15 on an existing trauma registry. The code set differences between AIS08 and AIS15 were known to be comparatively minor, compared with those between AIS98 and AIS08 [31]. However, changes in real-world datasets often differ in type and extent to those seen between AIS revisions, as there is considerable variation in the incidence of particular codes [26]. As such, it is notable that there were no significant differences in in-hospital mortality between AIS08-based and AIS15-based ISS when grouped into categories (Table S2), and very little difference across each individual ISS (Fig. 2). Although a slightly different ISS threshold (ISS08 ≥ 11 and ISS15 ≥ 12) provided the best comparability with an AIS98-based ISS ≥ 16 threshold, in practice this only affected 31 patients, and changed the number of patients classified as major trauma by less than 1%.

Defining major trauma using an ISS ≥ 16 has been regarded as standard since the 1980s [13, 14]. This study demonstrated that in-hospital mortality rates as well as ICU admission rates differ significantly and substantially when using the ISS ≥ 16 threshold across different AIS revisions. Comparing a threshold of ISS98 ≥ 16 with thresholds of ISS08 ≥ 11 and ISS15 ≥ 12 results in equivalent in-hospital mortality and ICU admission rates (Table 2). This is in line with the findings of Palmer et al. [27], who used a validated mapping tool converting AIS98 to AIS08. The present study externally validates their findings using manually coded injuries. Both trauma registries are in high income countries with a relatively low percentage of penetrating injuries, which renders them comparable in terms of epidemiology.

VSTR criteria fitted on DTR SW data resulted in higher medians and broader quartiles for ISS98, ISS08 and ISS15. The two registries have different inclusion criteria; the VSTR excludes specific isolated injuries such as isolated hip fractures, closed limb fractures, facial injuries and smaller burn injuries. The inclusion criteria of the DTR are more general; trauma < 48 h prior to admission to the ED and subsequent hospital admission, transfer to a different hospital or death (excluding death on arrival) [29]. Both registries are therefore especially comparable for the higher segments of injury severity, and showed virtually no differences in in-hospital mortality risk above an ISS of 11 (Fig. 2).

Injury codes for hypothermia, asphyxia (suffocation), (near) drowning, electrical injuries and whole body (explosion-type) are represented in AIS revisions from 2005 onward, and were thus lacking for our AIS98-coded cohort. This potentially resulted in selection and misclassification bias. Using the abovementioned injury codes for the AIS98 cohort, 62 extra patients were included and 41 patients had ISS98 scores upgraded. This enabled us to accurately represent all subgroups across both study periods. This resulted in a larger in-hospital mortality rate for MT patients. Comparing in-hospital mortality at alternative MT ISS thresholds for AIS08 and AIS15 compared with AIS98, the Breslow-D statistic displayed no homogeneity (Table S1). When adding the additional subgroups to the AIS98 cohort, homogeneity of ORs was present. Alternative ISS thresholds for MT increased to ISS ≥ 13 for both AIS08 and AIS15, compared to an ISS threshold of ≥11 and ≥ 12 respectively in a general trauma population described without the added injury codes.

Adjusted ISS thresholds for MT populations determined for use with AIS08- and AIS15-coded data provide ongoing comparability within trauma registries which have previously used AIS98, or across trauma registries using different AIS revisions. This assumes that the ISS remains an objective ‘gold standard’, instead of the de facto standard for measuring injury severity, assessing in-hospital mortality risk and providing quality indicators for measuring trauma network performance. However, this is not the case. Various studies have included other anatomical summary scores [11], or added physiological parameters and biomarkers [32, 33] to national trauma registries, and distinguish between severe single-system trauma and polytrauma [34, 35] to better define MT populations or risk-adjust when evaluating outcomes [36]. Modifying or selectively using ISS thresholds for defining MT should therefore be seen as an important, but temporary measure when comparing data collected across more than one AIS revision. In addition, growing interest in non-fatal functional outcomes like health-related quality of life and the evaluation of regionalization of trauma care, makes the definition of major trauma more layered and may give new insights the coming years.

### Strengths and limitations

Unlike other studies comparing the effects of different AIS revisions, the present study utilised AIS98 in one two-year period, and AIS08 (mapped to AIS15) in a second two-year period. An advantage of this methodology was that time-consuming double-coding, or potentially inaccurate mapping were avoided, making the study more easily replicable.

Although the time periods used were epidemiologically similar (Table 1), some differences may have remained. For example, within the DTR SW, patients primarily admitted to TC’s are known to have higher in-hospital mortality rates than patients admitted to non-trauma centers (NTC) with the same ISS. Consequently, the level of care could be a confounding variable for outcome due to case mix. This study only looked at the impact of a different AIS revisions from a regional point of view. Also, if patients were transferred between hospitals, primary registries of the referring hospital were excluded. Transfers to hospitals outside the DTR SW trauma region were not registered and not available for analysis. In spite of the similar proportions of transfers to the TC observed, some biases may have been present, either due to referral patterns, or injury coding differences between hospitals. Transferred patients are a complex subgroup due to the local health care context [37–39].

## Conclusion

When coding injuries using AIS08 or AIS15, thresholds of ISS08 ≥ 11 and ISS15 ≥ 12 respectively, perform similarly to a threshold of ISS ≥ 16 in AIS98 in terms of in-hospital mortality and ICU admission. After adjusting for non-codable injuries in AIS98, this threshold is ISS ≥ 13 for AIS08 and AIS15. This confirms previous work evaluating AIS08 with mapped datasets, and is the first to present an evaluation of the effects of AIS15 on trauma registry datasets. Defining major trauma using an appropriate ISS threshold is important for quality indicators, comparing datasets and adjusting for injury severity, but should not replace efforts to develop more appropriate major trauma definitions.

## Supplementary Information

**Additional file 1: Table S1.** Odds ratios for in-hospital mortality in MT patients using different AIS versions (AIS98, AIS08 and AIS15) and using different MT ISS thresholds. Comparisons are of AIS08 or AIS15 with AIS98. ISS, Injury Severity Score. **Table S2.** Crude in-hospital mortality numbers (rates) and odds ratios (with 95% CI) in ISS categories for AIS98, AIS08 and AIS15.

## Data Availability

The datasets generated and/or analysed during the current study are not publicly available due legislative arguments, but are available from the corresponding author on reasonable request.

## Abbreviations

AIS: Abbreviated Injury Scale
DNTR: Dutch National Trauma Registry
ED: Emergency Department
HEMS: Helicopter Emergency Medical Services
HCU: High Care Unit
ISS: Injury Severity Score
ICU: Intensive Care Unit
LOS: Length of Stay
TC: Level I trauma center
MT: Major Trauma
MV: Mechanical Ventilation
LOS MV: Mechancal Ventilation Days
MCU: Medium Care Unit
NTC: Non-level I trauma centers
STROBE: Strengthening the Reporting of Observational Studies in Epidemiology
DTR SW: Trauma Region Southwest Netherlands
VSTR: Victorian State Trauma Registry
