# Supplementary material for: The definition of major trauma using different revisions of the abbreviated injury scale
Source: Scand J Trauma Resusc Emerg Med. 2021 May 27;29:71. doi: 10.1186/s13049-021-00873-7 (PMC8162011; doi:10.1186/s13049-021-00873-7)
Supplement: Supplementary file 1 — Additional file 1: Table S1. Odds ratios for in-hospital mortality in MT patients using different AIS versions (AIS98, AIS08 and AIS15) and using different MT ISS thresholds. Comparisons are of AIS08 or AIS15 with AIS98. ISS, Injury Severity Score. Table S2. Crude in-hospital mortality numbers (rates) and odds ratios (with 95% CI) in ISS categories for AIS98, AIS08 and AIS15. [file 13049_2021_873_MOESM1_ESM.docx]

**Supplemental tables**

| **AIS Revision** | **MT threshold used** | **OR** | **95% CI** | **Breslow-D** |
| --- | --- | --- | --- | --- |
| AIS98 | ISS ≥ 16 | 12.23 | (9.86-15.17) |  |
| AIS08 | ISS ≥ 16 | 16.98 | (14.02-20.56) | 0.025^●¥^ |
| AIS15 | ISS ≥ 16 | 16.54 | (13.66-20.02) | 0.040^●¥^ |
| AIS98 | ISS ≥ 16 | 12.23 | (9.86-15.17) |  |
| AIS08 | ISS ≥ 11 | 9.95 | (8.26-11.99) | 0.155^Ø¥^ |
| AIS15 | ISS ≥ 12 | 9.96 | (8.27-11.99) | 0.155^Ø¥^ |

●No homogeneity of OR

Ø Homogeneity of OR

¥ Cochran-Mantel-Haenszel p < 0.0001

**Table S1**. Odds ratios for in-hospital mortality in MT patients using different AIS versions (AIS98, AIS08 and AIS15) and using different MT ISS thresholds. Comparisons are of AIS08 or AIS15 with AIS98. ISS, Injury Severity Score.

|  | **In-hospital mortality** | | | | | | | |
| --- | --- | --- | --- | --- | --- | --- | --- | --- |
|  | **AIS98** | **AIS08** | **AIS15** | **AIS revision^*^** | **AIS98 compared with AIS08** | | **AIS08 compared with AIS15** | |
| **ISS** | **n** *(%)* | **n** *(%)* | **n** *(%)* | **Χ^2^** *(p)* | **Χ^2^** *(p)* | **OR** *(95% CI)* **^^^** | **Χ^2^** *(p)* | **OR** *(95% CI)* **°** |
| **1-3** | 10 *(0,5)* | 24 *(0,5)* | 12 *(0,5)* | 0.237 (*0.888)* | 0.095 *(0.758)* | 0.890 *(0.425,1.865)* | 0.206 *(0.650)* | 1.174 *(0.586,2.353)* |
| **4-8** | 24 *(0,3)* | 50 *(0,7)* | 57 *(0,6)* | 9.926 *(0.007)* | 9.885 *(0.002)* | 2.147 *(1.318,3.498)* | 0.884 *(0.347)* | 0.833 *(0.569,1.220)* |
| **9-11** | 169 *(2,1)* | 188 *(2,9)* | 190 *(2,8)* | 13.541 *(0.001)* | 10.904 *(0.001)* | *1.423 (1.153,1.756)* | 0.038 *(0.845)* | *0.980 (0.798,1.202)* |
| **12-14** | 5 *(0,9)* | 4 *(0,7)* | 8 *(1,2)* | 0.708 *(0.702)* | 0.105 *(0.746)* | *0.805 (0.215,3.013)* | 0.665 *(0.415)* | *1.644 (0.492,5.489)* |
| **16-24** | 42 *(5,0)* | 37 *(5,5)* | 37 *(5,3)* | 0.165 *(0.921)* | 0.155 *(0.694)* | 1.096 *(0.695,1.727)* | 0.014 *(0.906)* | 0.972 *(0.608,1.555)* |
| **25-40** | 88 *(21,3)* | 135 *(32,1)* | 134 *(32,1)* | 13.905 *(0.001)* | 10.944 *(0.001)* | 1.710 *(1.243,2.354)* | 0.000 *(0.983)* | 0.997 *(0.740,1.342)* |
| **41-75** | 29 *(35,4)* | 47 *(53,4)* | 47 *(54,0)* | 7.217 *(0.027)* | 5.335 *(0.021)* | 2.078 *(1.113,3.878)* | 0.007 *(0.933)* | 1.026 *(0.562,1.875)* |
| **Total** | 367 *(1,9)* | 485 *(2,4)* | 485 *(2,4)* | 13.646 *(0.001)* | 10.698 *(0.001)* | 1.257 *(1.096,1.441)* | 0.000 *(1.000)* | 1.000 *(0.880,1.136)* |

**Table S2**. Crude in-hospital mortality numbers (rates) and odds ratios (with 95% CI) in ISS categories for AIS98, AIS08 and AIS15.
